# 16S rRNA Gene Amplicon Profiling of the New Zealand Parasitic Blowfly Calliphora vicina

**DOI:** 10.1128/MRA.00289-21

**Published:** 2021-05-06

**Authors:** Nikola Palevich, Paul H. Maclean, Luis Carvalho, Ruy Jauregui

**Affiliations:** a AgResearch Limited, Grasslands Research Centre, Palmerston North, New Zealand; Indiana University, Bloomington

## Abstract

Here, we present a 16S rRNA gene amplicon sequence data set and profiles demonstrating the bacterial diversity of larval and adult Calliphora vicina, collected from Ashhurst, New Zealand (May 2020). The three dominant genera among the adult male and female *C. vicina* blowflies were *Serratia* and *Morganella* (phylum *Proteobacteria*) and *Carnobacterium* (phylum *Firmicutes*), while the larvae were also dominated by the genera *Lactobacillus* (phylum *Firmicutes*).

## Abstract (precis)

Here, we present a 16S rRNA gene amplicon sequence data set and profiles demonstrating the bacterial diversity of larval and adult Calliphora vicina, collected from Ashhurst, New Zealand (May 2020). The three dominant genera among the adult male and female *C. vicina* were *Serratia* and *Morganella* (phylum *Proteobacteria*) and *Carnobacterium* (phylum *Firmicutes*), while the larvae were also dominated by the genera *Lactobacillus* (phylum *Firmicutes*).

## ANNOUNCEMENT

Ectoparasitic flies (blowflies) are a significant animal welfare and production issue for farmers worldwide (1). Control of blowflies is problematic because the flies are unpredictable and highly mobile, and strike (or myiasis) is difficult to see initially but has an immediate impact on animal production and welfare. Currently, control relies heavily on the prophylactic application of long-acting chemicals to all sheep, but this approach is increasingly under threat due to the development of resistance to current treatments (2, 3). Calliphora vicina NZ_CalVic_NP (4, 5) was selected for microbiome assessment as a representative of a New Zealand field strain of *C. vicina*. In this study, we investigated the larval and adult male and female bacterial microbial profiles of *C. vicina* for the future development of new interventions such as probiotics, bioactive compounds, vaccines, or insecticides.

The *C. vicina* specimen larvae were collected from a farm site in the Ashhurst area of New Zealand (40°18′S, 175°45′E). Lab-reared blowflies were maintained on beef liver as the protein source and a 10% sugar solution, with the procedures for blowfly propagation and sample preparation based on those of Dear (6). To remove surface adherent bacteria from lab-reared *C. vicina*, pools of larvae and entire adult males and females were separated and washed twice in sterile phosphate-buffered saline (PBS; pH 7.4), snap-frozen in liquid nitrogen, and transferred to −80°C storage prior to DNA extraction. High-molecular-weight genomic DNA was isolated from *C. vicina* pooled samples of 100 larvae as well as 10 entire adult males and females per replicate (*n* = 5 for each), using a modified phenol-chloroform protocol recently applied to difficult samples (7–13). A DNA library was prepared using the Illumina (San Diego, CA) 16S V3 and V4 rRNA library preparation method according to the manufacturer’s instructions (14) and sequenced on the Illumina MiSeq platform with the 2 × 250-bp paired-end (PE) reagent kit v2, producing a total of 3,017,007 PE raw reads.

**FIG 1:**
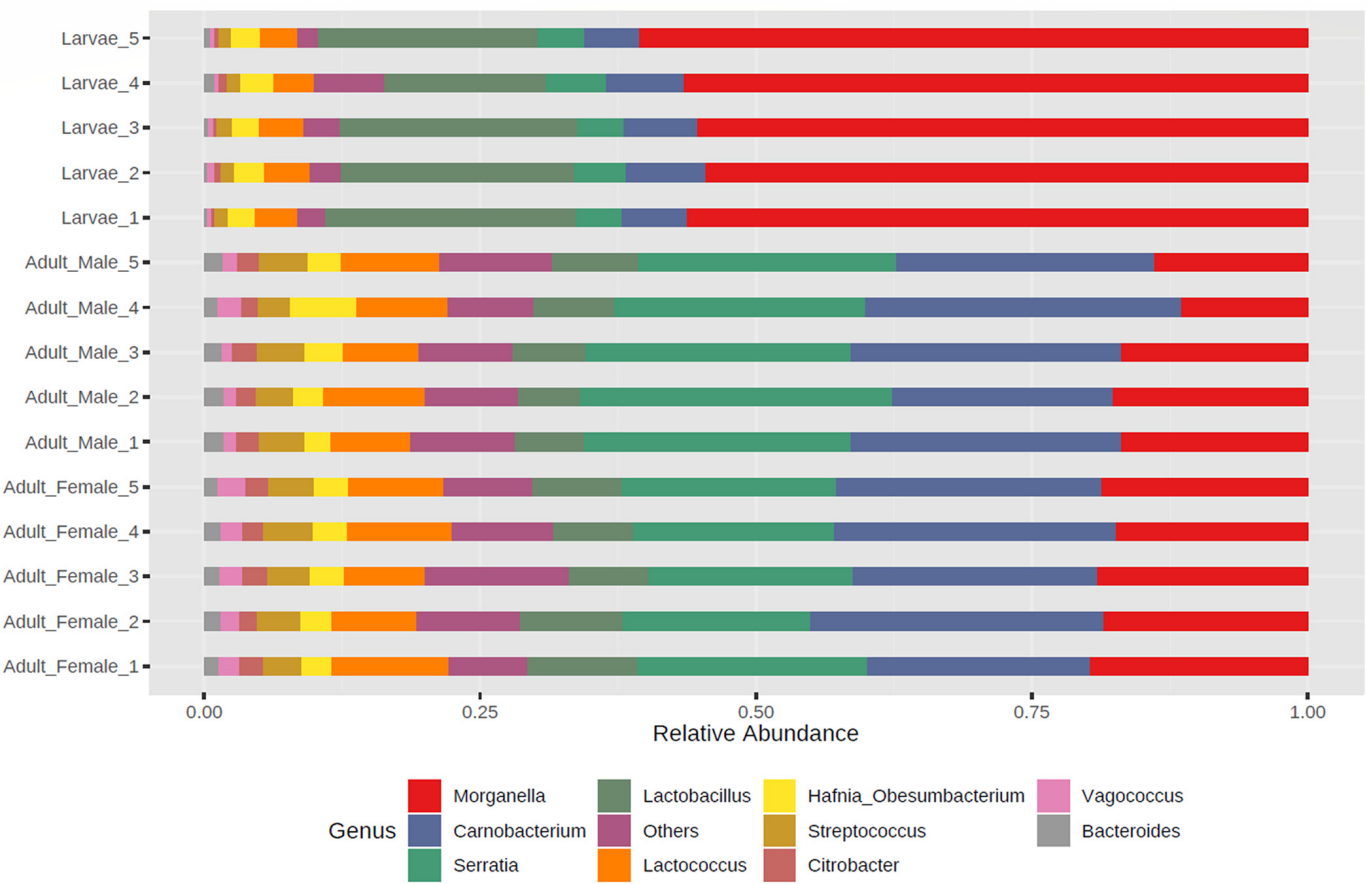
Taxonomic composition of the dominant bacteria of New Zealand *C. vicina*. The relative abundance of the dominant bacterial genera was obtained from 16S rRNA sequencing of *C. vicina* field strain NZ_CalVic_NP larvae and adult male and female samples. Genera with a relative abundance of less than 1% and unassigned amplicon sequence variants were grouped together as “others.”

The processing of the amplicon reads followed a modified version of the pipeline described in reference 15. Default parameters were used for all software unless otherwise specified. The reads produced by the sequencing instrument were paired using the program FLASH2 v2.2.00 (16). The paired reads were then quality trimmed using Trimmomatic v0.38 (17). The trimmed reads were reformatted as fasta files, and the read headers were modified to include the sample name. Mothur v1.45.2 (18) was used to remove reads with homopolymers longer than 10 nucleotides (nt) and to collapse the reads into unique representatives. The collapsed reads were clustered using Swarm v2 (19). The clustered reads were filtered based on their abundance, keeping representatives that were (i) present in one sample with a relative abundance of >0.1%, (ii) present in >2% of the samples with a relative abundance of >0.01%, or (iii) present in 5% of the samples at any abundance level (Fig. 1). The selected representatives were annotated using Qiime 2 v2017.4 (20) with the Silva database v138 (21). The metagenomic 16S rRNA gene amplicon sequencing of *C. vicina* field strain NZ_CalVic_NP reported here is a valuable resource for future studies investigating the bacterial genetic mechanisms associated with flystrike.

### Data availability.

The 16S rRNA gene amplicon sequence data have been deposited in the GenBank Sequence Read Archive (SRA) under the BioProject accession number PRJNA667961.
